# Transforming Diabetes Care Through Artificial Intelligence: The Future Is Here

**DOI:** 10.1089/pop.2018.0129

**Published:** 2019-05-30

**Authors:** Irene Dankwa-Mullan, Marc Rivo, Marisol Sepulveda, Yoonyoung Park, Jane Snowdon, Kyu Rhee

**Affiliations:** ^1^ IBM Corporation, Watson Health, Bethesda, Maryland.; ^2^ Population Health Innovations, Inc., Miami Beach, Florida.; ^3^ Occupational Medicine, University of Iowa, Iowa City, Iowa.; ^4^ IBM Corporation, IBM Research, Cambridge, Massachusetts.; ^5^ IBM Corporation, Watson Health, Yorktown Heights, New York.; ^6^ IBM Corporation, Watson Health, Cambridge, Massachusetts.

**Keywords:** diabetes care, artificial intelligence, cognitive computing, artificial pancreas, retinal imaging, glucose monitoring

## Abstract

An estimated 425 million people globally have diabetes, accounting for 12% of the world's health expenditures, and yet 1 in 2 persons remain undiagnosed and untreated. Applications of artificial intelligence (AI) and cognitive computing offer promise in diabetes care. The purpose of this article is to better understand what AI advances may be relevant today to persons with diabetes (PWDs), their clinicians, family, and caregivers. The authors conducted a predefined, online PubMed search of publicly available sources of information from 2009 onward using the search terms “diabetes” and “artificial intelligence.” The study included clinically-relevant, high-impact articles, and excluded articles whose purpose was technical in nature. A total of 450 published diabetes and AI articles met the inclusion criteria. The studies represent a diverse and complex set of innovative approaches that aim to transform diabetes care in 4 main areas: automated retinal screening, clinical decision support, predictive population risk stratification, and patient self-management tools. Many of these new AI-powered retinal imaging systems, predictive modeling programs, glucose sensors, insulin pumps, smartphone applications, and other decision-support aids are on the market today with more on the way. AI applications have the potential to transform diabetes care and help millions of PWDs to achieve better blood glucose control, reduce hypoglycemic episodes, and reduce diabetes comorbidities and complications. AI applications offer greater accuracy, efficiency, ease of use, and satisfaction for PWDs, their clinicians, family, and caregivers.

## Background

Diabetes is a global pandemic. An estimated 425 million people worldwide have diabetes, accounting for 12% of the world's health expenditures, and yet 1 in 2 persons remain undiagnosed and untreated.^1^ Type 2 diabetes is driven by the global obesity epidemic and a sedentary lifestyle that overwhelms the body's internal glucose control requiring exogenous insulin.^2^ Millions of newborns are born to mothers with gestational diabetes. Children born with type 1 diabetes mellitus, in which the body cannot produce insulin, require life-long insulin therapy. In the United States, diabetes is the leading cause of kidney failure, lower limb amputations, adult-onset blindness, and almost doubles the risk of heart attack and all-cause mortality, leading to hospitalization, long-term complications, and higher costs.^3^

Decades of well-designed studies have established that intensive therapy effectively delays the onset and slows the progression of diabetes-related complications, such as retinopathy, nephropathy, and neuropathy.^4^ Yet, a recent study of 300,000 patients with type 2 diabetes who were started on medical therapy found that after 3 months, 31% of patients had discontinued their diabetes medications altogether: this increased to 44% by 6 months, and to 58% by 1 year. Only 40% eventually restarted diabetes medications.^5^ Optimal care for persons with diabetes (PWDs) often is hampered by the absence of real-time, key health information necessary to make informed choices associated with intensive therapy and tight diabetes control. Although advances in technology offer unprecedented and inexpensive access to essential information for many individuals in many fields, its impact in the care of patients with diabetes seems rather limited. The challenges of real-time diabetes care information are compounded by the rapid expansion of medical knowledge. The index of biomedical literature contains more than 28 million articles as of June 2018 and is growing at a rate of more than 850,000 new citations each year.^6^ Each person will generate more than 1 million gigabytes of health-related data in his or her lifetime, the equivalent of about 300 million books. An estimated 80% of health data is unstructured. This includes clinician notes, clinical trials, hospital records and discharge summaries, imaging and laboratory reports, and nonclinical data sources, including device and sensor data (often referred to as Internet of Things data), genomic data, and social determinants of health data.^7^ Ninety percent of a person's health outcomes may be attributed to genomics and exogenous data, underscoring the importance of PWDs and their clinicians collecting and leveraging these data to make informed health choices.^8^

Rapid advances in artificial intelligence (AI) offer the promise of making both real-time structured and unstructured health data available for the care of PWDs. The Turing Archive for the History of Computing defines AI as “the science of making computers do things that require intelligence when done by humans.”^9^ AI covers a broad range of approaches to simulating human intelligence and performing various reasoning tasks, such as visual perception, speech recognition, analytics, decision making, and translation between languages. Cognitive systems employ the spectrum of AI approaches to extend and scale human knowledge and expertise by enabling humans to leverage vast knowledge sources rapidly to solve problems.

Today, AI is harnessing massive amounts of vital information to meet consumer demand in every business, including health care. A 2017 survey found that 68% of mobile health app developers and publishers believe that diabetes continues to be the single most important health care field with the best market potential for digital health solutions within the near future, and that 61% see AI as the most disruptive technology shaping the digital health sector.^10^ Although advances in AI for health care are being reported in the literature^11^ and new AI-powered devices are being approved for diabetes care,^12^ a systematic review of clinically relevant diabetes AI applications is missing. The purpose of this article is to better understand what meaningful AI advances may be relevant today to PWDs, their primary care clinicians, endocrinologists, health professionals, family, and caregivers.

## Methods

The study team conducted a predefined, online PubMed search of publicly available sources of information using the search terms “diabetes” and “artificial intelligence (AI).” To identify articles with clinically-relevant, high-impact diabetes AI applications, the team excluded manuscripts with publication dates before 2009 and those whose purpose was primarily technical in nature (eg, focused solely on AI algorithm development). The first-pass search identified a total of 763 clinically-relevant abstracts. Additional review excluded 313 as duplicative or primarily technical. The second-pass review yielded a total of 450 unique, clinically-relevant articles researching the direct application of AI in diabetes prevention, diagnosis, and treatment. The information was then collated and classified. The research was conducted between March and May of 2018.

## Results

The PubMed search yielded a total of 450 clinically-relevant and high-impact articles published in the last decade related to the field of applied AI in diabetes care. The AI applications aimed to improve a broad spectrum of diabetes care, from diabetes screening and detection to monitoring and treatment, and included apps, devices, and systems that aid patients, clinicians, and health systems. The published articles included in this search were of high clinical impact in that they sought to produce and test AI approaches that may impact diabetes care significantly in the areas of access, accuracy, efficiency, affordability, speed, and satisfaction of patients, clinicians, and caregivers. A review of the high-impact articles suggests that AI applications are aiming to transform diabetes care in 4 main areas: automated retinal screening, clinical decision support, predictive population risk stratification, and patient self-management tools, as summarized in Table 1.

**Table 1.** Categorization of Artificial Intelligence and Diabetes Care

*Category*	*Number of articles*	*Most common clinical AI applications*
Automated Retinal Screening	96	Detection of diabetic retinopathy, maculopathy, exudates, and other abnormalities from normal findings
Clinical Decision Support	126	Detection and monitoring of diabetes and comorbidities such as neuropathy, nephropathy and wounds
Predictive Population Risk Stratification	135	Identification of diabetes subpopulations at higher risk for complications, hospitalization, and readmissions
Patient Self-Management Tools	94	AI-improved glucose sensors, artificial pancreas, activity and dietary tracking devices
TOTAL	450	

AI, artificial intelligence.

A diverse and complex set of AI approaches and cognitive computing systems were employed in these studies. Table 2 defines the more common AI approaches described in the research and lists their clinical applications in diabetes care.

**Table 2.** Common Artificial Intelligence Approaches Used in Diabetes Care

*Method*	*How it works*	*Strengths*	*Limitations*	*Application area*
Multilayer perceptron	Composed of neurons in input layer, output layer, and multiple hidden layers. Neurons in each layer are connected to all neurons in the next layer, making each layer fully connected to the next. Learns by “backpropagation” method	Can model complex nonlinear relationship	Greater number of parameters have to be estimated without convolution Less effective than many other deep models	Prediction models, patient self-management tools
Convolutional neural network (CNN)	Composed of multiple layers of neurons with the convolution layer having neurons that look at small patches of the input image at a time, like a filter, and are convolved across the whole input image and share parameters across the image. Learns by “backpropagation” method Each layer of the CNN detects the presence of specific features across space, detecting more high-level features as moving forward	Can model complex nonlinear relationship Ideal for image, audio, video	Require a large amount of data to train Computationally intensive Many parameters require fine tuning while training the model	Retinal screening
Random forest	Creates an ensemble of decision trees In each tree, a random set of features are considered for determining root nodes and splits	Easy to fit, generally produces good performance Can be used for both classification and regression problems Can measure relative importance of features easily Robust to outliers and avoids overfitting, given sufficient sample size	Can be slow in prediction Only applicable for discrete outcomes - if outcome is continuous, it must be categorized Difficult to interpret	Retinal screening, decision support, prediction models, patient self-management tools
Fuzzy logic/fuzzy system	Provides a probability value between 0 and 1 rather than deterministic decision (0 or 1) for membership in a certain class	Resembles human reasoning High interpretability Easy to modify rules Does not require large data	Requires an expert curation of rules	Retinal screening, decision support, sensors and artifical pancreas
Support vector machine (SVM)	Classification method for binary outcomes (not often used for multiclass problems, but techniques for multiclass SVM exist) Works by adding data to a high-dimensional space and finds a hyperplane that separates 2 classes best (that maximizes the distance between the plane and nearby data points, or margin)	Performs well in nonlinear decision boundaries Robust to overfitting	Does not scale well to large data Hard to interpret	Retinal screening, decision support, prediction models, patient self-management tools
Logistic regression	Classification method for binary outcome Predicts the probability of an outcome (0 or 1) based on the features Learns the model coefficients by maximum likelihood estimation Finds a line or hyperplane that best represents the data points	Easy to fit, efficient, and scalable Supported by most standard software Can obtain probability of the outcome, which can be useful	Only binary classification Can be sensitive to outliers Requires transformation of nonlinear features	Prediction models
Natural language processing	Computational tools and methods to process, analyze, and perform inference of human languages	Critical in building intelligent machine and human–computer interactions Can process and analyze free-text information such as electronic physician notes	Usually requires a large amount of human-annotated records to train	Prediction models
K-nearest neighbors algorithm	Categorizes input data into several classes using its k nearest neighbors	Does not make assumptions about underlying distribution Can be used for both classification and regression problems Easy to understand and implement	Computationally intensive Sensitive to outliers or localized data	Retinal screening, decision support, prediction models, patient self-management tools

A review of the published articles documented the substantial advances in AI technology over the last 10 years and how it is helping PWDs and their clinicians make more informed choices. Examples of the most common AI-powered diabetes care devices and systems identified in the published literature are summarized in Table 3. Table 3 provides examples of the research questions receiving the most attention among diabetes AI researchers and product developers over the past decade.

**Table 3.** Summary of Selected Key Diabetes Artificial Intellegence Studies and Description of Outcomes

*Author, date*	*Title*	*Learning model*	*Training data/validation data/features*	*Testing data/features*	*Study outcomes*	*Model performance application*
**Retinal Screening**						
Gulshan V. 2016^18^	Development and Validation of a Deep Learning Algorithm for Detection of Diabetic Retinopathy in Retinal Fundus Photographs	Deep CNN	Data set: 128,175 retinal images Ground truth: images graded 3 to 7 times for DR, diabetic macular edema, and image gradability by a panel of 54 US licensed ophthalmologists	Test data: EyePACS-1 data set (n = 9963 images from 4997 patients) Messidor-2 data set (n = 1748 images from 874 patients)	Sensitivity: 97.5% for EyePACS-1, 96.1% for Messidor-2 Specificity: 93.4% for EyePACS-1, 93.9% for Messidor-2 ROC: 0.991 (95% CI, 0.988–0.993) for EyePACS-1, 0.990 (95% CI, 0.986–0.995) for Messidor-2	Deep machine learning algorithm had high sensitivity and specificity for detecting referable DR.
Rahim S. 2014^20^	Detection of Diabetic Retinopathy and Maculopathy Using Fuzzy Image Processing	Fuzzy image processing, ML classifiers (1-nearest neighbour, NB, SVM)	Dataset: public data sets (DIARETDB0, DIARETDB1, MESSIDOR, DRIVE, STARE, REVIEW, ROC) New data set of 600 images from 300 patients in Malaysia - oversampled to a total of 990 images Training data: 90% of data set	Test data: remaining 10% data	For k-NN, polynomial SVM, RBF SVM, and NB, respectively Sensitivity: 0.87, 0.45, 0.92, 0.91 Specificity: 1.00, 0.98, 0.94, 0.75 Accuracy: 0.93, 0.7, 0.93, 0.75	Fuzzy image processing together with the retinal structure extraction in DR screening can help produce a more reliable and efficient screening system
Lam C. 2018^21^	Retinal Lesion Detection With Deep Learning Using Image Patches	CNN (GoogLeNet)	Data set: manually created image patches from public image data set (Kaggle retinopathy data subset, n = 243) Ground truth: labeled by 2 ophthalmologists Training data:1050 patches Validation: 274 patches	Test data: public image data set (eOphta) (n = 463) Ground truth: labeled by 2 ophthalmologist at pixel level (3 classes)	In validation using the patch images: Accuracy 98%, ROC 99% with GoogLeNet For microaneurysm, exudates respectively in test data: Pixel-level ROC: 0.94, 0.95 Precision and recall AUC for detection task: 0.86, 0.64	Regionally trained CNNs can detect and distinguish between subtle pathologic lesions with only a few hundred training examples per lesion.
Keel S. 2018^23^	Feasibility and Patient Acceptability of a Novel Artificial Intelligence-based Screening Model for Diabetic Retinopathy at Endocrinology Outpatient Services	Deep CNN (Inception v3)	Data set: public data set (LabelMe, n = 66,790) Ground truth: Images graded by 21 ophthalmologists Training data: 58,790/66,790 Validation: 8000/66,790	Test data: data from 96 participants who agreed to receive both retinal screening approaches and complete a questionnaire Ground truth: compared to manual screening result by ophthalmologist	Sensitivity: 92.3% Specificity: 93.7% 96% of participants reported that they were either satisfied or very satisfied with the automated screening model 78% reported that they preferred the automated model over manual.	AI-based DR screening appears to be feasible, accurate, and well accepted by patients attending endocrinology outpatient settings.
**Predictive Modeling and Risk Stratification**						
Han L. 2015^25^	Rule Extraction from Support Vector Machines Using Ensemble Learning Approach: An Application for Diagnosis of Diabetes	Ensemble learning using SVM and RF rule extraction	Data set: China Health and Nutrition Survey data (n = 7913, 646 diabetic) Training data: 90% of dataset Validation: 10-fold cross-validation for model parameter selection 15 features selected using univariate LR, chi-square tests, information gain-based method, and RF	Test data: remaining 10% of data	For positive cases: Precision: 89.6% Recall: 44.3% F-score: 0.593 For all cases: Weighted average precision: 94.2% Weighted average recall: 93.9%	The proposed hybrid system can provide a tool for the diagnosis of diabetes from population-based nutritional surveys, and it supports a second opinion for lay users
Shankaracharya. 2012^26^	Computational Intelligence-based Diagnosis Tool for the Detection of Prediabetes and Type 2 Diabetes in India	Mixture of expert system based on MLP	Data set: 1415 subjects (947 diabetic) Training data: 1104/1415	Test data: 311/1415	Best result achieved Sensitivity: 99.5% Specificity: 99.07% Accuracy: 99.36%	The proposed tool for identifying individuals with prediabetes, diabetes, and nondiabetes is highly accurate and may be used for large-scale diabetic screenings.
Wei WQ. 2010^27^	A High Throughput Semantic Concept Frequency Based Approach for Patient Identification: A Case Study Using Type 2 Diabetes Mellitus Clinical Notes	NLP, SVM, and semantic knowledge	Data set: 57,707 electronic notes from 1600 DM patients and 1600 control patients in Mayo Clinic Validation: 10-fold cross-validation for model selection Features: Semantic concept units extracted from notes and classified into semantic type groups	No separate test data were specified	F-score for cases: 0.956 F-score for controls: 0.957 Precision for cases: 0.968. Semantic knowledge: varying degrees of F-score, precision, and recall values reported.	The proposed approach is accurate and responsive to the urgent need to develop a general automatic approach for diabetic patient case-finding and characterization.
Corey KE. 2016^28^	Development and Validation of an Algorithm to Identify Nonalcoholic Fatty Liver Disease (NAFLD) in the Electronic Medical Record	LR with adaptive LASSO	Data set: electronic medical records from 620 patient randomly selected from the high-risk patients in Partners Healthcare Ground truth: compared to chart review by a hepatologist Features: laboratory measurements, diagnosis codes, and concepts extracted from medical notes	Test data: randomly selected 611 high-risk patients identified by classification algorithm Additional validation: independent test set of 314,292 patients Ground truth: 100 random positive case record review	Specificity; 91% Sensitivity: 51% PPV: 89% NPV: 56% AUC: 0.85 (compared to 0.75 using ICD-9 billing codes only, *P* < 0.0001)	The NAFLD classification algorithm is superior to ICD-9 billing data alone. This approach is simple to develop, deploy, and can be applied across different institutions to create EMR-based cohorts of individuals with NAFLD.
Neves J. 2015^29^	A Soft Computing Approach to Kidney Diseases Evaluation	Logic Program-ming, ANN	Data set: data from 558 total patients (175 diagnosed with CKD) Training data: 2/3 of data set Clinical information about CKD as rewritten into Logic Programming algorithms, and its terms as training and test sets of ANN Features: 24 variables grouped into 5 categories	Test data: remaining 1/3 of data	ANN performance in test data set Sensitivity: 93.19% Specificity: 91.9% PPV: 84.4% NPV: 96.6%	The proposed model showed good performance in predicting the likelihood of a CKD diagnosis
Rau HH. 2016^30^	Development of a Web-based Liver Cancer Prediction Model for Type II Diabetes Patients by Using an Artificial Neural Network	ANN, LR	Data set: data from 2060 diabetic patients in the National Health Insurance Research Database (NHIRD) of Taiwan Training data: 1442/2060	Test data: 618/2060	ANN performance was superior to that of LR for predicting diabetics who will be diagnosed with liver cancer in the next 6 years. Sensitivity: 0.757 Specificity: 0.755 AUC: 0.873	Data mining systems enable clinicians to predict those diabetics at greater risk for the development of liver cancer.
Vyas R. 2016^32^	Building and Analysis of Protein-Protein Interactions Related to Diabetes Mellitus Using Support Vector Machine, Biomedical Text Mining and Network Analysis	SVM	Training data: positive and negative proteins from PDB and UniProt databases (n = 2653)	Test data: 129 proteins extracted via text mining from literature	Accuracy: 78.20% Precision: 68.26% AUC: 0.788	This integrated approach has a potential to identify disease-related proteins, functional annotation, and other proteomics studies.
López B. 2018^33^	Single Nucleotide Polymorphism (SNP) Relevance Learning with Random Forests for Type 2 Diabetes Risk Prediction	Random forest, k-NN	Data set: data from 677 subjects (248 diabetic), each containing 96 SNPs regarding type 2 diabetes Features: SNP data, clinical information, SNP value relevance	Test data: 10-fold cross-validation used. No separate test data were specified	For risk prediction AUC: 0.89 RF outperformed SVM and LR in terms of prediction accuracy and stability of the estimated relevance	RF is a useful method for learning predictive models to help physicians to identify the relevant SNPs associated with and predictive of type 2 diabetes.
**Clinical Decision Support**						
Lo-Ciganic WH. 2015^34^	Using Machine Learning to Examine Medication Adherence Thresholds and Risk of Hospitalization	Random survival forests, survival trees models	Data set: 33,130 non-dual-eligible Medicaid enrollees with type 2 diabetes Training data: 90% of data set Features: sociodemographics, measures of service use, health status, diabetes treatment intensity	Test data: remaining 10% data	The adherence thresholds most discriminating for risk of all-cause hospitalization varied from 46% to 94% - the widely used 80% adherence threshold is not optimal for predicting risk of hospitalization	Machine learning approaches hold promise as an intuitive and powerful approach for customizing interventions in medication adherence in diabetics and optimizing health outcomes.
Shu T. 2017^35^	An Extensive Analysis of Various Texture Feature Extractors to Detect Diabetes Mellitus Using Facial Specific Regions	k-NN, SVM with 8 image extractor methods	Data set: 284 diabetes mellitus and 231 healthy samples	Test data: 10-fold cross-validation used. No separate test data were specified	The best texture feature extractor, Image Gray-scale Histogram (bin n = 256), combined with SVM Sensitivity: 99.64% Specificity: 98.26% Accuracy: 99.02%	Compared with traditional diagnostic methods that rely on blood samples, the Image Gray-scale Histogram is a highly accurate, non-invasive way to diagnose diabetes using facial and tongue features.
Katigari KM. 2017^36^	Fuzzy Expert System for Diagnosing Diabetic Neuropathy	Fuzzy expert system	Data set: diagnostic parameters and their importance developed by specialists used to develop fuzzy expert system	Test data: 213 medical records of patients diagnosed with diabetic neuropathy	For diagnosis and severity of diabetic neuropathy Sensitivity: 89% Specificity: 98% Accuracy 93%	The fuzzy expert system can help diagnose and determine the severity of diabetic neuropathy.
Wang L. 2017^37^	Area Determination of Diabetic Foot Ulcer Images Using a Cascaded Two-Stage SVM-Based Classification	Two-stage SVM with simple linear iterative clustering and conditional random fields	Data set: 100 foot ulcer images from 15 patients	Test data: cross-validation used. No separate test data were specified	Sensitivity: 73.3% Specificity = 94.6%	Computer-based systems provide high performance rates for measuring diabetic wounds and monitoring wound healing status, and are sufficiently efficient for smartphone-based image analysis.
**Glucose Sensors and Artificial Pancreas**						
Mauseth R. 2015^38^	Testing of an Artificial Pancreas System With Pizza and Exercise Leads to Improvements in the System's Fuzzy Logic Controller	Fuzzy Logic Controller systems (FLC)	N/A	Total 17 meal, 13 exercise studies in 10 subjects with type 1 diabetes (T1D) FLC v2.0 test: 9 meal and 4 exercise studies with FLC v2.0, followed by interim analysis FLC v2.1 test: remaining 8 meal and 9 exercise studies using updated FLC	FLC v2.1 showed improvements in mean blood glucose after pizza consumption, after exercise testing, in reducing hyperglycemia, and percentage time spent in euglycemic range	Stress testing the AP system followed by adjustments to the dosing matrix significantly improved FLC performance when retested for mean blood glucose, high blood glucose, and normal blood glucose
Ling SH. 2012^39^	Natural Occurrence of Nocturnal Hypoglycemia Detection Using Hybrid Particle Swarm Optimized Fuzzy Reasoning Model	Fuzzy reasoning model with hybrid particle swarm optimization with wavelet mutation	Data set: 16 type 1 diabetic patients Training data: 320 data points from 8/16 patients	Test data: remaining 269 data points from 8/16 patients	Advanced noctural hypoglycemic episode detection Sensitivity: 85.7% Specificity: 79.8% Hypoglycemic episodes detection Sensitivity: 80.0% Specificity: 55.1%	The proposed system offers a noninvasive means to detect hypoglycemic episodes in type 1 diabetic patients.
Herrero P. 2015^40^	Advanced Insulin Bolus Advisor Based on Run-To-Run Control and Case-Based Reasoning	Combination of R2R and CBR	N/A	In silico testing using commercial type 1 diabetes simulator generated 1-month data for 10 adults and 10 adolescents scenarios	Using CBR(R2R), mean blood glucose improved in both adult and adolescent populations and hypoglycemia was completely eliminated (R2R alone was not able to do it in the adolescent population)	The proposed smartphone system keeps the simplicity of a standard bolus calculator while enhancing its performance by providing more adaptability and flexibility.
DeJournett L. 2016^41^	In Silico Testing of an Artificial-Intelligence-Based Artificial Pancreas Designed for Use in the Intensive Care Unit Setting	Knowledge-based system	N/A	In silico analysis: 126 000 unique 5-day simulations resulting in 107 million glucose values	On average, time in control range was 94.2%, time in range 70–140 mg/dl was 97.8%, time in hyperglycemic range was 2.1%, time in hypoglycemic range was 0.09% Average coefficient of variation: 11.1%	An AI-based closed-loop glucose controller may be able to improve on the results achieved by currently existing ICU-based PID/MPC controllers
**Patient Diabetes Self-Management Tools**						
Zhang W. 2015. ^44^	“Snap-n-Eat”: Food Recognition and Nutrition Estimation on a Smartphone	SVM	Data set: 2000 food images comprising 15 predefined categories Ground truth: manual annotation	Test data: 5-fold cross validation	Accuracy: 85%	The proposed smartphone mobile system can recognize food items present on a plate and estimates their calorific and nutrition content, automatically helping diabetic patients make more informed food choice decisions.
Cvetković B. 2016^45^	Activity Recognition for Diabetic Patients Using a Smartphone	Ensemble of models (SVM, J48, random forest, Jrip, AdaBoost and Bagging algorithms), symbolic rules	Data set: average 11 hours of phone and 7.5 hours of ECG recordings per day for 2 weeks from 9 healthy volunteers Training data: first week of recordings Features (if present): sound, location, acceleration, heart-rate, respiration-rate	Test data: second week of recordings	Best result achieved by Multi-Classifier Adaptive Training (MCAT) method Accuracy: 83.4% F-score: 0.82	Smartphone sensors using machine learning and symbolic reasoning can recognize and quantify high-level lifestyle activities of diabetic patients and help them make more informed activity choices.
Wang L. 2015^46^	Smartphone-based Wound Assessment System for Patients with Diabetes	Image boundary detection: mean-shift segmentation algorithm Color segmentation: K-means clustering	N/A	30 simulated wound images, 34 actual patient wound images	Visual evaluation for simulated images Matthews Correlation Coefficient: 0.736	The proposed smartphone camera system enables diabetic patients and their caregivers to take a more active role in daily wound care.
Rigla M. 2018^47^	Gestational Diabetes Management (GDM) Using Smart Mobile Telemedicine	Mobile telemedicine system	NA	20 patients diagnosed with GDM (Parallel observational prospectively captured clinical data for historical control)	Metabolic and perinatal outcomes were similar except for BP, which was lower in patients using the telemedicine system	Artificial-intelligence-augmented telemedicine has been proposed as a helpful tool to facilitate an efficient widespread medical assistance to GDM.

AI, artificial intelligence; ANN, artificial neural network; AP, artificial pancreas; AUC, area under the curve; BP, blood pressure; CBR, case-based reasoning; CKD, chronic kidney disease; CI, confidence interval; CNN, convolutional neural network; DM, diabetes mellitus; DR, diabetic retinopathy; ECG, electrocardiogram; EMR, electronic medical record; FLC, Fuzzy Logic Controller; GDM, gestational diabetes management; ICD-9, *International Classification of Diseases, Ninth Revision*; k-NN, k nearest neighbors algorithm; LASSO, least absolute shrinkage and selection operator; LR, logistic regression; MLP, multilayer perceptron; MPC, model predictive control; N/A, not applicable; NA; NAFLD, nonalcoholic fatty liver disease; NB, naïve bayes; NLP, natural language processing; NPV, negative predictive value; PDB, protein databank; PID, proportional integral derivative; PPV, positive predictive value; R2R, run-to-run; RBF, radial basis function; RF, random forest; ROC, receiver operating characteristic; SNP, single nucleotide polymorphism; SVM, support vector machine.

## Discussion

The published studies suggest that a broad spectrum of market-ready AI approaches are being developed, tested, and deployed today in the prevention, detection, and treatment of diabetes. The total number of published technical articles reporting advances in the field of diabetes and AI increased exponentially in the past decade, from 2600 in 2008, to 5500 in 2013, to more than 10,000 in 2017.^13^ Millions of patient health records and newly published research exist that need to be further processed, analyzed, and learned from to create a current diabetes knowledge base for patients, researchers, doctors, and clinicians. Because of AI's ability to rapidly interpret and process enormous amounts of data into simple actionable guidance, these published studies suggest that AI has significant potential to improve screening, diagnosis, and management of patients with diabetes.^14^

Researchers are employing various AI approaches to interpret the vast amount of relevant data that need to be analyzed and assessed.^15^ Table 2 describes the more common AI approaches described in the research and lists their clinical applications in diabetes care. AI involves a wide spectrum of increasingly complex algorithms encompassed within the terminology of machine learning, deep learning, and cognitive computing. In machine learning, experts typically “train” AI systems with large amounts of data and algorithms, which enable the machine to examine relationships and learn from them. In deep learning, AI systems identify relevant insights for diagnostic support, while automatically conducting certain complex and time-consuming tasks. Cognitive AI systems go even further by *understanding, reasoning, interacting*, and *learning*. These systems understand by processing and deeply interpreting the available data, both structured and unstructured, at enormous speed and volume. They reason by understanding entities and relationships, making connections, proposing hypotheses, and evaluating evidence. In contrast to the electronic health record, they provide a more natural interaction between human and computer, facilitating dialogue, visualization, and collaboration.^16^ Cognitive AI Systems learn by collecting and evaluating feedback at all levels of the system. The result is practical knowledge, aids, and devices for diabetes patients and their clinicians that save time, improve efficiency, enhance clinical decision making, empower patients, and have the potential to improve health outcomes and patient and clinician satisfaction. Although a more complete explanation of AI is beyond the focus of this research article, this study indicates that AI is a growing presence in diabetes care with the potential to transform millions of people's lives.

Today, research suggests that AI approaches are rapidly transforming care in 4 vital areas: improved screening and detection of diabetic retinopathy (DR) and macular edema; individualized predictive risk stratification and treatment; decision-support tools for clinicians; and patient self-management aids. Key examples from the published literature are summarized in Table 3 and follow.

DR is the most serious cause of secondary blindness, exacting an enormous burden on individuals, families, and the health care system. The annual diabetic retinal exam serves to screen and proactively detect diabetes patients with early treatable retinopathy. It is estimated that 98% of vision loss from DR and macular edema is avoidable through improved prediction, early detection, and treatment strategies, and its cost-effectiveness is well established.^17^ Yet, major barriers to implementing more widespread screenings include the limited number of eye care practitioners who are trained in interpreting retinal images, along with access to care barriers.

Today, research documents that deep learning-based AI-grading of DR from retinal photographs is associated with sensitivity and specificity over 90%.^18–21^ Recently, the US Food and Drug Administration (FDA) approved marketing the first medical device to use AI to screen diabetes patients for retinopathy.^6^ The device, called IDx-DR (IDx LLC, Coralville, IA), is a software program that uses an AI algorithm to analyze images of the eye taken with a retinal camera called the Topcon NW400 (Topcon Medical Systems, Inc., Oakland, NJ). Digital images of the patient's retinas are uploaded to a cloud server on which IDx-DR software is installed. If the images are of sufficient quality, the software provides the doctor with one of 2 results: (1) “more than mild diabetic retinopathy detected: refer to an eye care professional” or (2) “negative for more than mild diabetic retinopathy; rescreen in 12 months.” IDx-DR is the first device authorized for marketing that provides a screening decision without the need for a clinician to also interpret the image or results.^22^ These automatic systems enable non–eye health professionals in primary care physician offices to perform on-site retinal screening and provide on-the-spot normal results or immediate referrals to the eye specialist without the need for eye specialists, with significantly higher patient satisfaction with the simplified process.^23,24^

Today, AI-driven predictive modeling proactively identifies diabetes populations with the highest risks of avoidable complications resulting in unnecessary emergency department visits, admissions, and readmissions.^25^ Larger physician groups, health care systems, and health plans utilize AI to “mine” large sets of digital and unstructured patient data to proactively identify and characterize diabetes populations,^26,27^ find patients at risk for diabetic comorbidities,^28–30^ identify patients for special diabetes disease management programs,^31^ and discover relevant proteins^32^ and genes^33^ associated with and predictive of diabetes.

Today, AI provides practice decision-support tools for physicians and other health professionals caring for PWDs. Machine learning approaches help physicians customize diabetes medications to optimize adherence and health outcomes.^34^ AI-powered devices help physicians diagnose diabetes noninvasively,^35^ and more accurately measure and monitor the severity of diabetic neuropathy^36^ and diabetic wounds.^37^

Today, research suggests that diabetes management for both PWDs and their clinicians is being simplified and improved by new sensors, pumps, smartphone applications, and other breakthroughs in AI to achieve better blood glucose control,^38^ reduce hypoglycemic episodes,^39^ and improve patient satisfaction and reported outcomes.^40^ A 2017 meta-review of published clinical trials of the latest, automated, personal or real-time continuous glucose monitoring devices (RT-GCM) using computerized AI algorithms concluded that a wide range of AI-powered RT-CMG devices are entering the market to enable PWDs and their clinicians to assess and improve glycemic control, reduce hypoglycemic episodes, especially at night, and to improve A1c levels.

Published research documents the extensive testing under way with the “artificial pancreas,” known also as a Closed Loop System, which combines continuous glucose measurement with algorithm-driven insulin pumps to reduce hypoglycemia and improve diabetes self-care.^41^ The latest-generation sensors, which are more accurate and sensitive for hypoglycemia, and the development of algorithms that allow insulin infusion to be suspended during hypoglycemia and glucagon to be administered, provide a safe and effective system for persons at high risk of hypoglycemia. A meta-review of 12 published transition and home studies of 10 to 58 patients comparing clinical performance and patient acceptance of Artificial Pancreas Devices (APDs) with traditional monitoring concluded that research, testing, and validation has moved from the laboratory to free-living, unsupervised home settings in the past decade, with accuracy and reliability of the latest APD devices compatible with safe operation and high patient satisfaction.^42^ A recent review identified 18 closed-loop APDs being tested – 6 APDs in the home setting, 5 in outpatient settings, and 7 in inpatient settings – with planned commercial availability in 2018 and 2019.^43^

Today, research findings document the promise of diabetes apps to assist users in tracking and analyzing their data in a hassle-free way and to deliver personalized data-driven insights that PWDs may apply in their daily life. Today, best-in-class apps provide comprehensive nutrition databases that tell a user the nutritional content after scanning the barcode, allow them to search for restaurant menu items or popular meals by their names, or recognize food items on a plate.^44^ Smartphone sensors using machine learning and symbolic reasoning can recognize and quantify high-level lifestyle activities of patients with diabetes and help them make more informed activity choices.^45^ An AI smartphone camera system enables patients with diabetes and their caregivers to take a more active role in daily wound care, and may potentially accelerate wound healing, save travel cost, and reduce health care expenses.^46^ AI-augmented telemedicine has been studied to facilitate medical assistance in the homes of pregnant women with gestational diabetes, with a high degree of patient acceptance. A research study incorporated computer-interpretable clinical practice guidelines, and access to data from the electronic health record as well as from glucose, blood pressure, and activity sensors.^47^

On March 14, 2018, the FDA approved Medtronic's Guardian Connect, the first AI-powered continuous glucose monitoring (CGM) system, for use in PWDs between the ages of 14 and 75 years. Guardian Connect utilizes a predictive algorithm that alerts patients of significant swings in blood glucose levels up to 60 minutes prior to the event. When combined with the Guardian Sensor 3, which is placed on the abdomen to monitor blood glucose levels every 5 minutes and sent to a personal app, the Guardian Connect system was accurate and was able to alert patients of about 98.5% of hypoglycemic events so that they could proactively take action to normalize blood sugar.^48^

This information also can be shared and monitored with caretakers and family members in real time or via text message. In addition, Guardian Connect CGM is connected to the Sugar.IQ smart diabetes assistant. Utilizing AI technology from IBM Watson Health, the Sugar.IQ assistant continually analyzes how a patient's blood glucose levels respond to factors such as food intake, insulin dosages, and daily routines. Relative to baseline metrics, Sugar.IQ conferred in 256 Guardian Connect users tested a 36-minute/day improvement in blood glucose time-in-range or 9 full days a year, a 30-minute/day decrease in time >180 mg/dl, and a 6-minute/day decrease in time <70 mg/dl, all statistically significant. During the course of the 31+ patient-years of use, Sugar.IQ generated 655 insights for PWDs related to hypoglycemia and 699 related to hyperglycemia. In addition, 134 Sugar.IQ users were randomly given Fitbits exercise monitoring applications during the course of the study. Results showed that glucose responses to meals and activity vary greatly, demonstrating the importance of personalization in diabetes self-management. Notably, 231 of the 256 (90%) users recorded at least 2 weeks of data, demonstrating a solid pattern of engagement with the AI-powered diabetes self-management application.^49^

Many challenges remain before diabetes AI apps, devices, and systems become ubiquitous in the health care marketplace. One major challenge is technical interoperability between systems: the ability of 2 or more systems to exchange and use the information.^50^ In addition, expensive up-front and ongoing costs, physician cooperation, and the complexity of meeting Meaningful Use criteria stifle adoption and innovation.^51^

Another major challenge is the limitation in reproducing AI results from published studies. A most basic problem is that researchers often do not share their source codes, sometimes for competitive reasons. A survey of 400 algorithms presented in papers at 2 top AI conferences in the past few years revealed that only 6% of the presenters shared the algorithm's code. Only one third shared the data used to test their algorithms, and just half shared the “pseudocode”–a limited summary of a source code algorithm.^52^ In addition, assuming one can obtain and run the original pseudocode, it still might not do what is expected. In the area of AI called machine learning, in which computers derive expertise from experience, the training data for an algorithm – for example, the key information to train speech-recognition learning systems – also can influence its performance.

Despite these challenges, this review of recently-published, high-impact, and clinically-relevant studies suggests that diabetes is attracting top health care technology companies as well as start-ups that are using innovative AI technologies and approaches to tackle daily challenges faced by PWDs. Many of the applications have received regulatory approval in the past few years and are on the market today. Many more are on the way with the aim to disrupt and transform diabetes care by improving accuracy, efficiency, ease of use, simplicity, and enjoyment on behalf of PWDs and their providers, caregivers, and family. The published literature suggests that the combination of continuous monitoring and real-time feedback to PWDs may be able to identify meaningful patterns and lead to personalized insights that increase patient and clinician engagement, confidence, and success in maintaining blood glucose levels under better control.

## References

[1] International Diabetes Federation (IDF). IDF diabetes atlas, 7th edition. Brussels, Belgium: International Diabetes Federation, 2015

[2] Liu J, Wang R, Ganz ML, Paprocki Y, Schneider D, Weatherall J The burden of severe hypoglycemia in type 2 diabetes. Curr Med Res Opin 2018;34:179–186 29017368 10.1080/03007995.2017.1391080

[3] Centers for Disease Control and Prevention. Estimates of diabetes and its burden in the United States. Washington, DC: US Department of Health and Human Services, 2017:1–20

[4] The Diabetes Control and Complication Trial Research Group. The effect of intensive treatment of diabetes on the development and progression of long-term complications in insulin-dependent diabetes mellitus. N Engl J Med 1993;329:977–986 8366922 10.1056/NEJM199309303291401

[5] Latts L ADA/IBM Watson Health Study (N>300,000) finds that nearly 60% of people with T2D discontinue therapy after one year. Presented at: American Diabetes Association 78th Scientific Session, 6 22–26, 2018, Orlando, FL

[6] National Center for Biotechnology Information; US National Library of Medicine. PubMed. https://www.ncbi.nlm.nih.gov/pubmed/ Accessed 718, 2018

[7] Lewis K Humans and systems: creating natural interfaces to augment human ability. https://www.ibm.com/blogs/internet-of-things/creating-natural-interfaces/ Accessed 718, 2018

[8] Aggarwal M, Madhukar M IBM's Watson analytics for health care: a miracle made true. Hershey, PA: IGI Global, 2016:117–134

[9] Turing Archive for the History of Computing. www.alanturing.net/turing_archive/archive/index/archiveindex.html Accessed 718, 2018

[10] Research 2 Guidance. Top 3 therapy fields with the best market potential for digital health apps. https://research2guidance.com/top-3-therapy-fields-with-the-best-market-potential-for-digital-health-apps/ Accessed 718, 2018

[11] Yeung S, Downing NL, Fei-Fei L, Milstein A Bedside computer vision—moving artificial intelligence from driver assistance to patient safety. N Engl J Med 2018;378:1271–1273 29617592 10.1056/NEJMp1716891

[12] Young K Newly approved software uses AI to improve diabetic retinopathy. https://www.jwatch.org/fw114063/2018/04/12/newly-approved-software-uses-ai-detect-diabetic Accessed 718, 2018

[13] Pfaffl MW A new mathematical model for relative quantification in real-time RT-PCR. Nucleic Acids Res 2001;29:45e 10.1093/nar/29.9.e45 PMC55695 11328886

[14] López B, Martin C, Viñas PH Special section on artificial intelligence for diabetes. Artif Intell Med 2018;85:27–28 10.1016/j.artmed.2017.09.008 28943334

[15] Raghupathi W, Raghupathi V Big data analytics in healthcare: promise and potential. Health Inf Sci Syst 2014;2:3 25825667 10.1186/2047-2501-2-3 PMC4341817

[16] Hoyt RE, Snider D, Thompson C, Mantravadi S IBM Watson analytics: automating visualization, descriptive, and predictive statistics. JMIR Public Heal Surveill 2016;2:e157 10.2196/publichealth.5810 PMC5080525 27729304

[17] Vijan S, Hofer TP, Hayward RA Cost-utility analysis of screening intervals for diabetic retinopathy in patients with type 2 diabetes mellitus. JAMA 2000;283:889–896 10685713 10.1001/jama.283.7.889

[18] Gulshan V, Peng L, Coram M, et al. Development and validation of a deep learning algorithm for detection of diabetic retinopathy in retinal fundus photographs. JAMA 2016;316:2402–2410 27898976 10.1001/jama.2016.17216

[19] Tariq A, Akram MU, Shaukat A, Khan SA Automated detection and grading of diabetic maculopathy in digital retinal images. J Digit Imaging 2013;26:803–812 23325123 10.1007/s10278-012-9549-4 PMC3705025

[20] Rahim SS, Palade V, Shuttleworth J, Jayne C Automatic screening and classification of diabetic retinopathy fundus images. In: Mladenov V, Jayne C, Iliadis L, eds. Engineering applications of neural networks. Communications in computer and information science. Vol 459 Basel, Switzerland: Springer Nature Switzerland AG, 2014:113–122

[21] Lam C, Yu C, Huang L, Rubin D Retinal lesion detection with deep learning using image patches. Invest Ophthalmol Vis Sci 2018;59:590–596 29372258 10.1167/iovs.17-22721 PMC5788045

[22] US Food and Drug Administration. FDA permits marketing of artificial intelligence-based device to detect certain diabetes-related eye problems. https://www.fda.gov/NewsEvents/Newsroom/PressAnnouncements/ucm604357.htm Accessed 718, 2018

[23] Keel S, Lee PY, Scheetz J, et al. Feasibility and patient acceptability of a novel artificial intelligence-based screening model for diabetic retinopathy at endocrinology outpatient services: a pilot study. Sci Rep 2018;8:4330 29531299 10.1038/s41598-018-22612-2 PMC5847544

[24] Matimba A, Woodward R, Tambo E, Ramsay M, Gwanzura L, Guramatunhu S Tele-ophthalmology: opportunities for improving diabetes eye care in resource- and specialist-limited Sub-Saharan African countries. J Telemed Telecare 2016;22:311–316 26407990 10.1177/1357633X15604083

[25] Han L, Luo S, Yu J, Pan L, Chen S Rule extraction from support vector machines using ensemble learning approach: an application for diagnosis of diabetes. IEEE J Biomed Health Inform 2015;19:728–734 24860043 10.1109/JBHI.2014.2325615

[26] Shankaracharya, Odedra D, Samanta S, Vidyarthi AS Computational intelligence-based diagnosis tool for the detection of prediabetes and type 2 diabetes in India. Rev Diabet Stud 2012;9:55–62 22972445 10.1900/RDS.2012.9.55 PMC3448174

[27] Wei W-Q, Tao C, Jiang G, Chute CG A high throughput semantic concept frequency based approach for patient identification: a case study using type 2 diabetes mellitus clinical notes. AMIA Annu Symp Proc 2010;2010:857–861 21347100 PMC3041302

[28] Corey KE, Kartoun U, Zheng H, Shaw SY Development and validation of an algorithm to identify nonalcoholic fatty liver disease in the electronic medical record. Dig Dis Sci 2016;61:913–919 26537487 10.1007/s10620-015-3952-x PMC4761309

[29] Neves J, Martins MR, Vilhena J, et al. A soft computing approach to kidney diseases evaluation. J Med Syst 2015;39:131 26310948 10.1007/s10916-015-0313-4

[30] Rau H-H, Hsu C-Y, Lin Y-A, et al. Development of a web-based liver cancer prediction model for type II diabetes patients by using an artificial neural network. Comput Methods Programs Biomed 2016;125:58–65 26701199 10.1016/j.cmpb.2015.11.009

[31] Weber C, Neeser K Using individualized predictive disease modeling to identify patients with the potential to benefit from a disease management program for diabetes mellitus. Dis Manag 2006;9:242–256 16893337 10.1089/dis.2006.9.242

[32] Vyas R, Bapat S, Jain E, Karthikeyan M, Tambe S, Kulkarni BD Building and analysis of protein-protein interactions related to diabetes mellitus using support vector machine, biomedical text mining and network analysis. Comput Biol Chem 2016;65:37–44 27744173 10.1016/j.compbiolchem.2016.09.011

[33] López B, Torrent-Fontbona F, Viñas R, Fernández-Real JM Single nucleotide polymorphism relevance learning with random forests for type 2 diabetes risk prediction. Artif Intell Med 2018;85:43–49 28943335 10.1016/j.artmed.2017.09.005

[34] Lo-Ciganic WH, Donohue JM, Thorpe JM, et al. Using machine learning to examine medication adherence thresholds and risk of hospitalization. Med Care 2015;53:720–728 26147866 10.1097/MLR.0000000000000394 PMC4503478

[35] Shu T, Zhang B, Yan Tang Y An extensive analysis of various texture feature extractors to detect diabetes mellitus using facial specific regions. Comput Biol Med 2017;83:69–83 28237906 10.1016/j.compbiomed.2017.02.005

[36] Katigari MR, Ayatollahi H, Malek M, Haghighi MK Fuzzy expert system for diagnosing diabetic neuropathy. World J Diabetes 2017;8:80–88 28265346 10.4239/wjd.v8.i2.80 PMC5320751

[37] Wang L, Pedersen PC, Agu E, Strong DiM, Tulu B Area determination of diabetic foot ulcer images using a cascaded two-wtage SVM-based classification. IEEE Trans Biomed Eng 2017;64:2098–2109 27893380 10.1109/TBME.2016.2632522

[38] Mauseth R, Lord SM, Hirsch IB, Kircher RC, Matheson DP, Greenbaum CJ Stress testing of an artificial pancreas system with pizza and exercise leads to improvements in the system's fuzzy logic controller. J Diabetes Sci Technol 2015;9:1253–1259 26370244 10.1177/1932296815602098 PMC4667297

[39] Ling SH, Nguyen HT Natural occurrence of nocturnal hypoglycemia detection using hybrid particle swarm optimized fuzzy reasoning model. Artif Intell Med 2012;55:177–184 22698854 10.1016/j.artmed.2012.04.003

[40] Herrero P, Pesl P, Reddy M, Oliver N, Georgiou P, Toumazou C Advanced insulin bolus advisor based on run-to-run control and case-based reasoning. IEEE J Biomed Health Inform 2015;19:1087–1096 24956470 10.1109/JBHI.2014.2331896

[41] DeJournett L, DeJournett J In silico testing of an artificial-intelligence-based artificial pancreas designed for use in the intensive care unit setting. J Diabetes Sci Technol 2016;10:1360–1371 27301982 10.1177/1932296816653967 PMC5094333

[42] Thabit H, Tauschmann M, Allen JM, et al. Home use of an artificial beta cell in type 1 diabetes. N Engl J Med 2015;373:2129–2140 26379095 10.1056/NEJMoa1509351 PMC4697362

[43] Trevitt S, Simpson S, Wood A Artificial pancreas device systems for the closed-loop control of type 1 diabetes: what systems are in development? J Diabetes Sci Technol 2016;10:714–723 26589628 10.1177/1932296815617968 PMC5038530

[44] Zhang W, Yu Q, Siddiquie B, Divakaran A, Sawhney H “Snap-n-Eat”: food recognition and nutrition estimation on a smartphone. J Diabetes Sci Technol 2015;9:525–533 25901024 10.1177/1932296815582222 PMC4604540

[45] Cvetković B, Janko V, Romero AE, Kafalı Ö, Stathis K, Luštrek M Activity recognition for diabetic patients using a smartphone. J Med Syst 2016;40:256 27722975 10.1007/s10916-016-0598-y

[46] Wang L, Pedersen PC, Strong DM, Tulu B, Agu E, Ignotz R Smartphone-based wound assessment system for patients with diabetes. IEEE Trans Biomed Eng 2015;62:477–488 25248175 10.1109/TBME.2014.2358632

[47] Rigla M, Martínez-Sarriegui I, García-Sáez G, Pons B, Hernando ME Gestational diabetes management using smart mobile telemedicine. J Diabetes Sci Technol 2018;12:260–264 28420257 10.1177/1932296817704442 PMC5851209

[48] ClinicalTrails.gov Adult accuracy study of the Elite 3 Glucose Censor (E3). https://www.clinicaltrials.gov/ct2/show/study/NCT02246582?term=NCT02246582&rank=1&sect=X0123456 Accessed 718, 2018

[49] Neemuchwala H Sugar.IQ improves time-in-range by 36 mins/day, time >180 by 30 mins/day, time <70 by 6 mins/day. Presented at: American Diabetes Association 78th Scientific Session, 6 22–26, 2018, Orlando, FL

[50] Benson T Principles of health interoperability HL7 and SNOMED, 2nd edition. London: Springer Verlag, 2012.

[51] Adler-Milstein J, DesRoches CM, Kralovec P, et al. Electronic health record adoption in us hospitals: Progress continues, but challenges persist. Health Aff (Millwood) 2015;34:2174–2180 26561387 10.1377/hlthaff.2015.0992

[52] Hutson TM Missing data hinder replication of artificial intelligence studies. www.sciencemag.org/news/2018/02/missing-data-hinder-replication-artificial-intelligence-studies? Accessed 718, 2018

